# Allergy to citrus juice

**DOI:** 10.1186/2045-7022-3-S3-P153

**Published:** 2013-07-25

**Authors:** T Bourrier, C Pereira

**Affiliations:** 1 Pediatric Pneumology and Allergy Department, GCS Hôpitaux Pédiatriques de Nice CHU Lenval, Nice, France

## Background

Mathieu was diagnosed with egg allergy when he was 7 months old with complete tolerance at age 3. He subsequently developed an asthma (mite and dog sensitization) that was controlled with inhaled corticosteroids. At age 4, he developed a severe anaphylactic reaction shortly after drinking a fresh fruit juice of carrot and lemon. He drank a homemade juice and after fifteen minutes he had nausea with intense abdominal pain, an asthma exacerbation with wheezing, followed by repeated vomiting and, one hour later, a generalized urticaria. The symptoms were severe and the boy was admitted to the emergency department: he was administered corticosteroids and systemic antihistamine but not epinephrine, and was continuously monitored for 24 hours. He fully recovered and was discharged.

## Methods

Allergic investigations were undertaken : prick tests (prick to prick), IgE assays (CAP-FEIA f208 Thermo-Fisher^®^).

## Results

The native prick tests were negative for carrot, lemon pulp and lemon skin, but positive for lemon seed (9/5 mm). Extension tests were secondarily performed for other citrus: lemon pulp, lemon skin, lime, orange and grapefruit. These tests were all negative but, unfortunately, the parents brought seedless fruits. A third evaluation was planned with fruits containing seeds and then the tests were positive for lemon seed (10/6 mm) and orange seed (10/4 mm). The test with grapefruit seed was negative. Specific IgE assay confirmed the sensitization for lemon (8 kUI/l) and ruled out the sensitization for carrot (<0.1 kUI/l).

An eviction diet for lemon and other citrus was initiated with an anaphylaxis emergency action plan. The boy was provided with an epinephrine auto-injector ANAPEN^®^. A challenge with seedless lemon juice was not proposed regarding the severity of the initial reaction.

## Conclusion

Citrus food allergy is uncommon albeit the consumption of citrus juice (in particular orange juice) is high worldwide. Few data, both clinical and biochemical, were retrieved from the literature regarding this allergy. Mild symptoms like an oral allergy syndrome are generally reported; anaphylaxis is rare. This clinical case suggest that the role of seed is probably underestimated.

## Disclosure of interest

None declared.

